# Sequential Enzymatic Bioprocessing of Protein-Rich Unhairing–Liming and Lime-Fleshing Tannery Wastewater for the Production of Amino Acid-Based Plant Biostimulants

**DOI:** 10.3390/molecules31183213

**Published:** 2026-09-11

**Authors:** Henoc Pérez-Aguilar, Víctor M. Serrano-Martínez, Carlos Ruzafa-Silvestre, Alberto Vico, María Dolores Romero-Sánchez

**Affiliations:** INESCOP, Footwear Technology Centre, Pol. Ind. Campo Alto, C/Alemania, 102, 03600 Elda, Alicante, Spain; vmserrano@inescop.es (V.M.S.-M.); cruzafa@inescop.es (C.R.-S.); avico@inescop.es (A.V.); mdromero@inescop.es (M.D.R.-S.)

**Keywords:** tannery wastewater, sequential enzymatic hydrolysis, protein hydrolysates, free amino acids, plant biostimulants, beamhouse effluents, waste recovery, circular bioeconomy

## Abstract

The recovery of bioactive compounds from industrial wastewaters is a key strategy for improving resource efficiency and reducing the environmental impact of high-load effluents. In this work, two protein-rich tannery wastewater streams with different origins and compositions, unhairing–liming wastewater and lime-fleshing wastewater, were comparatively evaluated as secondary raw materials for the production of amino acid-based protein hydrolysates with potential biostimulant activity. Both streams were characterised in terms of physicochemical composition, mineral content and amino acid profile, and subsequently subjected to a comparative sequential enzymatic hydrolysis approach using endo- and exo-proteolytic enzymes. Alcalase, followed by Pancreatin, was selected as the most effective enzymatic system for both streams, although different optimal enzyme loadings were required depending on the wastewater composition. For unhairing–liming wastewater, the optimal conditions were 1.5% Alcalase and 1.0% Pancreatin, yielding 70.76 ± 1.95% hydrolysate, 80.87 ± 1.98% protein recovery and 11.42 ± 1.03% free amino acids. For lime-fleshing wastewater, 0.7% Alcalase and 1.0% Pancreatin provided 98.42 ± 1.97% yield, 92.19 ± 1.92% protein recovery and 16.97 ± 0.98% free amino acids. The two hydrolysates showed differentiated amino acid profiles, reflecting the keratin- and collagen-derived nature of the original streams. Germination assays indicated a growth-stimulating effect of the hydrolysates, increasing seed growth by up to 20.7% for the unhairing–liming wastewater hydrolysate at 0.10% (*w*/*v*) and by up to 27.1% for the lime-fleshing wastewater hydrolysate at 0.07% (*w*/*v*). These results demonstrate that enzymatic hydrolysis can be an effective and environmentally friendly route for converting tannery wastewaters into value-added bio-based products for agricultural applications, while also highlighting the industrial relevance of adapting the enzymatic process to the origin, composition and protein accessibility of each wastewater stream.

## 1. Introduction

Global waste generation is increasing as a consequence of population growth, industrial expansion and intensified agricultural and livestock production. More than 10 billion tonnes of agricultural, forest, municipal and industrial by-products are generated annually, creating an urgent need for technologies capable of transforming residual streams into valuable resources [1,2]. In this context, the recovery of bio-based compounds from industrial wastewaters has become a key strategy within circular economy models, particularly when these effluents contain organic matter that can be converted into functional products [3,4]. Among the available waste recovery approaches, environmentally friendly technologies based on enzymatic processes are especially attractive because they can operate under mild conditions, reduce the use of harsh chemicals and generate products with higher added value [5,6].

The leather industry is a representative example of a sector in which a biological by-product is transformed into a high-performance material, but also one of the industrial activities associated with significant water use and the generation of complex waste streams. Hides and skins, mainly composed of collagen, are converted into leather through a sequence of mechanical and chemical operations [7,8]. Although this process produces a durable material, it also generates large volumes of wastewater and protein-rich residues [8,9]. UNIDO estimates a global input of approximately 10 million tonnes of wet-salted hides per year, while previous studies indicate that only around 150 kg of leather are obtained from 1000 kg of raw hides, with the remaining fraction being converted mainly into solid residues and effluents [8,9]. In parallel, FAO data indicate that approximately 6.9 million tonnes of wet-salted hides are processed annually, generating about 4.5 million tonnes of solid waste [7,9,10]. These figures highlight the relevance of developing recovery strategies for the proteinaceous streams generated during leather manufacture. However, the environmental burden of leather processing is not limited to solid residues, since most beamhouse and tanning operations are water-intensive and transfer a significant fraction of the organic and inorganic load into liquid effluents.

Water consumption is, therefore, one of the main environmental concerns in tanning. Conventional leather processing may consume between 15 and 50 m^3^ of water per tonne of raw hide, generating a comparable volume of wastewater [8,9]. For the processing of wet-salted hides into finished leather, typical water consumption values of 12–37 m^3^/t have been reported. When expressed per unit area of leather, this represents an average water demand of approximately 115–146 L/m^2^. These values are particularly relevant because reducing water consumption does not necessarily decrease the total pollutant load. Rather, it may concentrate the same amount of organic matter, salts, sulphides and suspended solids in a smaller effluent volume [8]. Therefore, water-saving strategies must be complemented by recovery-oriented technologies aimed at reducing environmental impact while valorising the valuable compounds present in tannery wastewaters.

Beamhouse operations are especially important from both environmental and technological perspectives. These stages prepare the hide before tanning and include soaking, unhairing–liming, fleshing, splitting, deliming and bating [8,9]. Among them, unhairing–liming is one of the most critical steps. Its purpose is to remove hair and epidermis, eliminate residual interfibrillary components, and open the collagen fibre structure. In this context, interfibrillary components refer to the non-collagenous substances located between collagen fibre bundles, including proteoglycans, glycosaminoglycans, soluble proteins, globular proteins, residual epidermal material and other mucopolysaccharide-rich compounds naturally present in the hide matrix. Their partial removal during unhairing–liming facilitates fibre opening, increases water uptake and improves the accessibility of the collagen network for subsequent processing. This operation is usually carried out under strongly alkaline conditions using lime and sulphide-based reagents, which promote hair degradation, swelling of the collagen matrix and partial solubilisation of proteinaceous material [8,9]. As a result, unhairing–liming wastewater contains a complex mixture of degraded keratin, collagen-derived fragments, soluble proteins, peptides, fats, calcium salts, sulphides and suspended solids. This stream is, therefore, not only a high-load wastewater, but also a potentially valuable source of recoverable protein compounds.

In addition to unhairing–liming wastewater, lime-fleshing wastewater represents another relevant protein-rich effluent generated during leather processing. Fleshing residues are produced when residual flesh and fat are mechanically removed from hides [9,11]. When this operation is carried out after liming, the resulting waste stream contains collagen-derived proteins, fats, lime, salts and suspended solids. Previous studies have reported that, from the leather waste generated during hide processing, fleshing may account for approximately 979,800 tonnes, associated with around 764,200 m^3^ of wastewater rich in protein, 117,600 tonnes of solid protein and 98,000 tonnes of fat [7,9]. These values show that lime-fleshing wastewater is not a marginal stream, but a quantitatively important source of proteinaceous matter that could be recovered and converted into high-value products.

Current treatment strategies for tannery wastewaters are mainly designed to remove pollutants and reduce the organic load before discharge. Technologies such as ultrafiltration, isoelectric solubilisation, electrocoagulation, precipitation and biological treatment have been investigated for protein-rich effluents [12,13,14,15,16]. However, many of these approaches focus on separating or eliminating the pollutant load rather than recovering nutrients or functional molecules [16,17]. Moreover, some processes may involve high operational costs, significant sludge generation or limited selectivity. For this reason, the development of sustainable technologies that recover protein from tannery effluents, instead of only treating them as waste, represents an important opportunity for cleaner leather processing.

Protein-rich wastewaters from the tanning sector are particularly interesting for the production of hydrolysed products with potential application as plant biostimulants. In recent years, biostimulants derived from organic residues and industrial by-products have attracted increasing attention because they can contribute to waste reduction, nutrient recovery and the development of renewable alternatives to conventional mineral fertilisers and synthetic crop inputs [18,19]. The European biostimulants market has been reported to reach 1.590 million euros in 2024 and is expected to grow to 2.340 million euros by 2029, reflecting the increasing demand for sustainable agricultural inputs [20]. Protein hydrolysates are especially relevant within this market because they may contain peptides and free amino acids capable of improving nutrient uptake, plant metabolism, chlorophyll production and tolerance to abiotic stresses [21,22,23,24].

The use of animal by-products and protein-rich wastewaters for biostimulant production also fits within the current European regulatory framework for fertilising products, provided that processing methods are adapted to the origin of the raw material and to the intended final application. Regulation (EU) 1009/2019 establishes rules for EU fertilising products and opens the possibility of using certain derived products as fertilisers or biostimulants when they do not pose risks to animal, plant or human health [25]. In this regard, previous work has highlighted that fleshing wastewater and meat and bone meal wastewater can be considered as raw materials for hydrolysis products intended for use as biostimulants or organic fertilisers [26,27,28,29]. This regulatory and technological context supports the exploration of protein-rich tannery effluents as secondary raw materials for value-added agricultural products.

Among the different conversion technologies available, enzymatic hydrolysis is a promising route for recovering protein from wastewaters and animal by-products. Compared with chemical or thermal hydrolysis, enzymatic hydrolysis can operate under milder pH and temperature conditions, offers greater selectivity and may reduce environmental impact [5,30]. Proteolytic enzymes cleave peptide bonds in protein substrates, promoting the formation of soluble peptides and free amino acids [5,31]. Previous studies have shown that enzymatic hydrolysis can be applied to different protein-rich matrices, including meat and bone meal wastewater, soaking flesh wastewater, lime-fleshing wastewater and other animal by-product streams [26,27,28,29]. These studies have demonstrated that enzymatic treatment can produce hydrolysates with relevant amino acid content and potential use as bio-based products, while also contributing to water reuse and wastewater load reduction.

Sequential enzymatic hydrolysis is especially suitable for complex and heterogeneous protein-rich wastewaters. This strategy usually combines proteases with complementary activities, such as an endoprotease followed by an exoprotease [26,27,28,29,32]. The first enzymatic step promotes the internal cleavage of protein chains, increasing substrate accessibility and generating peptide fragments, whereas the second step favours further degradation into low molecular weight peptides and free amino acids. This approach is particularly relevant for tannery effluents because their composition may vary strongly depending on hide origin, preservation method, beamhouse formulation, liming conditions, fleshing stage and separation efficiency. Parameters such as pH, ash content, fat content, suspended solids and protein concentration can strongly affect enzyme activity and hydrolysis performance.

Despite the potential of enzymatic hydrolysis, the literature focused on the direct recovery of protein from tannery wastewater through enzymatic processes is still limited. Most studies on tannery effluents are oriented towards wastewater treatment, pollutant removal, energy recovery or sludge reduction [12,13,14,15,16,17]. In contrast, fewer works approach these streams as a source of recoverable nutrients and bioactive compounds. This gap is particularly relevant for unhairing–liming and lime-fleshing wastewater, which is generated before the tanning stage and, therefore, contains proteinaceous material that has not been exposed to chromium salts or other tanning agents. Its comparison with lime-fleshing wastewater may provide useful information on how the origin and composition of the effluent influence enzymatic hydrolysis efficiency and the quality of the obtained hydrolysates.

In this study, two protein-rich tannery wastewaters are compared as secondary raw materials to produce amino acid-based hydrolysates: unhairing–liming wastewater and lime-fleshing wastewater. This comparison is relevant because both streams are generated during beamhouse operations but differ in origin, composition and expected protein accessibility. Unhairing–liming wastewater is a liquid effluent containing solubilised collagen- and keratin-derived compounds, sulphides, lime, fats and suspended solids. Lime-fleshing wastewater, in contrast, is associated with the mechanical removal of flesh and fat after liming and may contain a more concentrated mixture of collagen-derived proteins, fats, calcium salts and suspended matter. These compositional differences may influence the need for conditioning, enzyme accessibility, hydrolysis yield, amino acid release and final product quality.

The aim of this work is to develop and evaluate an environmentally friendly enzymatic process for the recovery of protein hydrolysates from unhairing–liming wastewater and lime-fleshing wastewater. Both effluents are first characterised in terms of their physicochemical composition, including pH, solid content, fat content, ash content and protein content. Subsequently, a sequential enzymatic hydrolysis process based on proteases with complementary activities is applied and optimised. The resulting hydrolysates are evaluated according to protein recovery, total and free amino acid content, physicochemical properties and potential suitability as plant biostimulants.

By comparing two relevant tannery wastewater streams, this study provides a broader assessment of enzymatic hydrolysis as a waste recovery technology for the leather sector. The proposed approach converts high-load proteinaceous effluents into value-added hydrolysates, contributing to wastewater valorisation, the reduction of the environmental burden and the development of renewable biostimulant products. This work is, therefore, aligned with environmentally friendly technologies for waste processing and recovery, offering a circular strategy that integrates tannery wastewater management, protein recovery and the production of bio-based agricultural inputs.

## 2. Materials and Methods

### 2.1. Materials

The proteolytic enzymes employed for the production of protein hydrolysates enriched in free amino acids were (A) ALC, Alcalase 2.5 L, an alkaline *endo*-protease with 2.5 AU-A/g activity, supplied by Univar Solutions Spain (Barcelona, Spain); (B) BPT, Bioprotease LA660, an alkaline endo-protease (850 UB/g); (C) PANCREATIN, a neutral *exo*-protease (600 IU/g) and (D) NP, ALLZYME NP, an alkaline *endo*-protease (190,000 LVU/g) from Spain Enzymes S.L. (Valencia, Spain). Potassium hydroxide and phosphoric acid were obtained from PANREAC (Castellar del Vallès, Spain).

The streams used in this study consisted of unhairing–liming and lime-fleshing wastewater provided by Industrias del Curtido S.A. (INCUSA), located in Silla (Valencia, Spain). These wastewaters are derived directly from the company’s leather production process. This aqueous fraction originates from the unhairing–liming and fleshing stage of the beamhouse and contains partially solubilised collagen and keratin, fats, salts, sulphides, and suspended solids. Prior to enzymatic hydrolysis, the wastewater was screened to remove coarse solids, ensuring a homogeneous and consistent substrate suitable for processing.

### 2.2. Methods

#### 2.2.1. Characterisation of Unhairing–Liming and Lime-Fleshing Wastewaters and Hydrolysates

Initial characterisation of the wastewater is essential to design an effective protein recovery [26,27,28,29]. Characterisation of the raw wastewater prior to hydrolysis is essential to implement an efficient protein recovery strategy. Parameters such as total solids, protein, fat, and ash content were determined to guide the conditioning and optimisation of the enzymatic process. When the fat content exceeded 5–6%, a pre-treatment step was applied to facilitate enzyme accessibility and maximise hydrolysis efficiency [5,28,29]. This assessment ensures that the resulting hydrolysates are enriched in free amino acids and suitable for bio-based applications.

Analytical determinations were performed according to international standards: pH (EN ISO 4045:2008), fat content (EN ISO 4048:2018), ash content (EN ISO 4047:1977), moisture (EN ISO 4684:2005), and heavy metal content (EN ISO 17072-2:2019) [33,34,35,36,37]. Total amino acids were quantified in compliance with ISO 13903:2005, involving derivatisation to form chromophores, followed by separation using liquid chromatography with fluorescence and diode-array detection [38]. Free amino acids were extracted with 0.01 N hydrochloric acid, and co-extracted macromolecules were precipitated with sulfosalicylic acid and removed by filtration [38]. For all measurements, three independent replicates were performed, and the results reported correspond to the mean of these replicates.

In this study, the total amino acid content determined after acid hydrolysis was used as an indicator of the protein-derived fraction and is reported throughout the manuscript as “protein content”. Protein recovery was calculated as the amount of protein-derived material recovered in the final hydrolysate relative to that initially present in the wastewater, taking into account the hydrolysate mass yield and its protein content.

#### 2.2.2. Recovery of Protein from Unhairing–Liming and Lime-Fleshing Wastewater

Protein recovery was achieved through a sequential two-step enzymatic hydrolysis designed to generate hydrolysates enriched in low molecular weight peptides and free amino acids (<10 kDa), consistent with regulatory requirements (Order APA/161/2020, modifying Spanish RD 506/2013) [39]. The first hydrolysis step employed an endo-protease (ALC or BPT) to cleave internal peptide bonds, followed by an exo-protease Pancreatin (PAN), to further degrade the peptides and release amino acids.

Hydrolysis was carried out in a 1 L glass-jacketed bioreactor equipped with a three-necked lid for a pH meter (Sension+ PH1, Hach, Ames, IA, USA), a thermometer, and a mechanical stirrer (RZR 2041, Heidolph, Nuremberg, Germany) operating at 450 rpm. The reactor jacket was connected to a thermostatic bath (Ultraterm-TFT-200, J.P. Selecta, Barcelona, Spain) to maintain the desired temperature; 300 mL of unhairing–liming wastewater was used for each hydrolysis run.

In the first step with ALC, pH was maintained at 8.5 using 2 N KOH and the temperature at 70 °C. When using BPT, the temperature was set at 55 °C with the same pH. Enzyme loads between 0.5 and 2.5% (*w*/*w*) were tested for 5 h. The second hydrolysis step was conducted at pH 7.5 using a 10% phosphoric acid solution and 45 °C with Pancreatin for 3 h.

**Figure 1:**
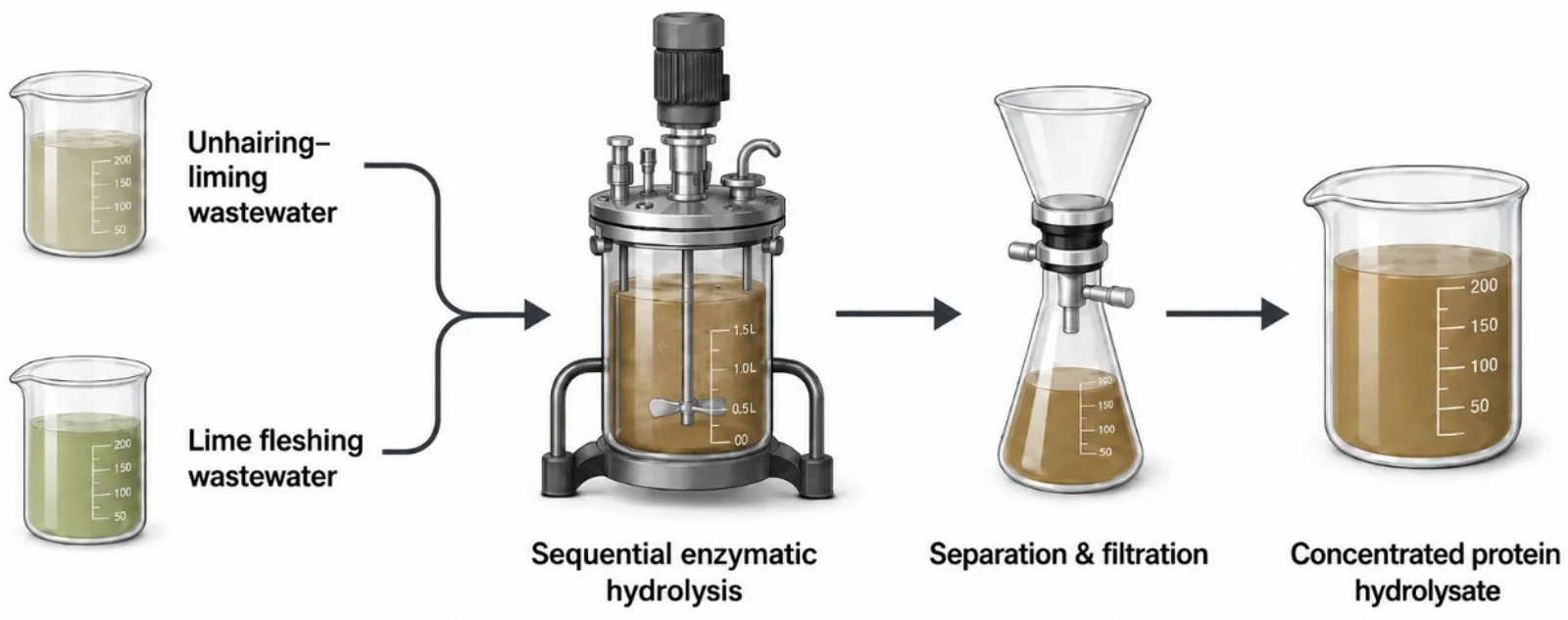
Process to obtain protein hydrolysate from tannery wastewater.

Following hydrolysis, the mixture was centrifuged at 3700 rpm for 5 min to separate unreacted solids, which were discarded. The supernatant, containing the protein hydrolysate, was subjected to thermal treatment at 122 °C for 21 min to ensure complete inactivation of the proteolytic enzymes. This step was included as a process-stopping treatment to prevent residual Alcalase or Pancreatin activity from continuing peptide bond cleavage during cooling, handling, storage or freeze-drying. Thermal inactivation is a standard approach in the production of protein hydrolysates, since residual protease activity may otherwise modify the degree of hydrolysis, molecular weight distribution and free amino acid content after the defined reaction time (Figure 1).

#### 2.2.3. Validation of the Hydrolysate as a Potential Biostimulant

The functional evaluation of the protein hydrolysates for use as a potential plant biostimulant was conducted using a germination-based growth assay following a methodology consistent with previous studies [26,27,28,29] and in compliance with EN ISO 16086-1 and EN ISO 16086-2:2012 (“Soil improvers and growing media–Determination of plant response–Part 1: Pot growth test with Chinese cabbage; Part 2: Petri dish test using cress”) [40,41]. This procedure is widely accepted for assessing the efficacy of hydrolysed protein with growth-stimulating activity in promoting early plant growth.

For each hydrolysate concentration, the germination assay was performed in triplicate. Each replicate consisted of one Petri dish containing 10 Chinese cabbage (*Brassica rapa pekinensis*, Batle) seeds. Therefore, a total of 30 seeds were evaluated for each hydrolysed protein dose. Each Chinese cabbage seed was placed on qualitative filter paper (Branchia), 70 mm in diameter and 10–15 µm pore size, characterised by very fast filtration, and positioned inside sterile 90 mm Petri dishes (Deltalab S.L., Rubí, Spain). A volume of 2 mL of hydrolysate solution, prepared at different concentrations ranging from 0.01 to 0.2% (*w*/*v*), was applied to each dish. The seeds were incubated at 24 °C for three days. Following this initial period, an additional 2 mL of the same hydrolysate solution was applied, and the seeds were maintained under identical conditions for a further three days.

After six days of incubation, seedling growth was evaluated by direct measurement of the germinated seedling length using a millimetre ruler. Each seedling was carefully removed from the Petri dish and placed on a flat surface without stretching or damaging the plant material. The total seedling length was measured individually from the point of emergence at the seed coat to the distal end of the developed radicle/seedling axis. Measurements were expressed in millimetres, and the same measurement criterion was applied to all treated samples and to the control group.

For each hydrolysate concentration, three independent Petri dishes were used, each containing 10 seeds. The Petri dish was considered the experimental unit, while the 10 seeds within each dish were treated as technical subsamples. Individual seedling lengths were used to calculate the mean seedling length for each Petri dish, and the treatment mean and standard deviation were obtained from the three independent dish-level values.

Seeds with a final length below 10 mm were initially considered as showing abnormal or incomplete germination and were excluded from the calculation of the average seedling length. This threshold was used as an operational criterion to avoid including seeds with very poor or irregular germination, which may arise from seed damage, lack of viability or abnormal development. Nevertheless, since reduced seedling growth may also represent a genuine inhibitory response to the treatment, the number of excluded seeds was recorded for each hydrolysate concentration and considered in the interpretation of the dose–response behaviour.

#### 2.2.4. Statistical Analysis

Data obtained from amino acid quantification and germination assays were analysed using STATGRAPHICS Centurion v19 software (Statgraphics Technologies, The Plains, VA, USA). One-way or multifactor analysis of variance (ANOVA) was applied according to the number of experimental factors included in each dataset. The measured response, such as the total amino acid content, free amino acid content or seed germination length, was considered the dependent variable, whereas the hydrolysate type, enzymatic treatment and concentration were included as categorical independent variables, as applicable.

Statistical significance was evaluated at a 95% confidence level (*p* < 0.05). When significant effects were identified by ANOVA, differences among treatment means were evaluated using Fisher’s Least Significant Difference (LSD) post-hoc test at the same significance level. Fisher’s LSD was selected because pairwise comparisons were conducted only after a significant overall ANOVA and involved a predefined and limited number of treatments within balanced experimental designs with replicated measurements.

## 3. Results and Discussion

### 3.1. Characterisation of Unhairing–Liming Wastewater and Lime-Fleshing Wastewater

Enzymatic hydrolysis is a selective and controllable process for protein depolymerisation, in which pH, temperature and stream composition strongly influence the efficiency of peptide bond cleavage. In the present study, two protein-containing aqueous streams from the beamhouse stage of leather processing were evaluated: unhairing–liming wastewater and lime-fleshing wastewater. Both streams originate from alkaline operations prior to tanning and, therefore, contain proteinaceous matter that has not been exposed to tanning agents. However, they differ significantly in origin and composition, which may affect enzyme accessibility, hydrolysis efficiency and the composition of the final hydrolysates.

Unhairing–liming wastewater was collected from Industrias del Curtido S.A. (INCUSA, Silla, Valencia, Spain) and corresponds to the aqueous effluent generated during hair removal and hide opening. This wastewater contains partially solubilised keratin from hair, collagen-derived fragments, residual fats, inorganic salts, sulphides and suspended solids. Lime-fleshing wastewater, in contrast, is associated with the fleshing operation carried out after liming, where residual flesh and fat are mechanically removed from hides. This stream is expected to contain a higher proportion of collagen-derived material, together with fats, lime-derived salts and suspended proteinaceous matter. Therefore, the comparative characterisation of both effluents is essential to determine their suitability as streams for enzymatic hydrolysis and their potential for producing amino acid-rich hydrolysates with growth-stimulating activity.

Table 1 summarises the proximate composition of both wastewater streams. The two effluents showed marked differences in pH, solid content, protein concentration and mineral fraction. Unhairing–liming wastewater presented a strongly alkaline pH of 9.63, consistent with the use of lime and sulphide-based reagents during hair removal. In contrast, lime-fleshing wastewater showed a lower pH value of 6.15, which can be attributed to the previous neutralisation steps applied after liming. This difference is relevant because pH directly affects enzyme activity and determines the need for pH adjustment prior to hydrolysis.

The solid content of lime-fleshing wastewater was considerably higher than that of unhairing–liming wastewater, with values of 11.08% and 4.67%, respectively. This result is consistent with the nature of the fleshing operation, which generates an aqueous stream enriched in suspended and partially solubilised organic matter derived mainly from residual flesh and collagen-rich tissue. In contrast, unhairing–liming wastewater is a more diluted stream, although it still contains suspended and colloidal proteinaceous fragments derived from keratin degradation, epidermal residues and partial collagen solubilisation.

The difference between both streams was especially evident in their total amino acid content. Lime-fleshing wastewater showed a total amino acid content of 9.10% in the liquid stream and 82.13% on a dry basis, indicating a high concentration of recoverable proteinaceous matter. This can be explained by the partial hydrolysis of collagen during liming, which favours the transfer of protein fragments into the aqueous phase. In comparison, unhairing–liming wastewater contained 1.55% total amino acids in the liquid stream and 33.2% on a dry basis. Although lower than that observed for lime-fleshing wastewater, this protein content is still relevant because the stream is generated in large volumes and contains keratin- and collagen-derived compounds that can be enzymatically converted into soluble peptides and free amino acids.

The fat content of both streams was relatively low when expressed in the aqueous phase, with values of 0.35% for unhairing–liming wastewater and 0.73% for lime-fleshing wastewater. On a dry basis, these values corresponded to 7.49% and 6.59%, respectively. These levels are not expected to significantly interfere with enzyme–substrate interactions. In enzymatic hydrolysis processes, high lipid contents may reduce efficiency due to emulsification phenomena or the formation of micellar structures that hinder enzyme access to protein substrates [5,26,27,28,29]. However, the fat levels measured in both streams were moderate, suggesting that direct enzymatic hydrolysis can be performed without requiring an intensive defatting stage.

Although the ash content of unhairing–liming wastewater appears high when expressed on a dry matter basis, 47.41%, this value should be interpreted together with the low solid content of the original stream. The wastewater contained only 4.67% solids and 95.33% moisture. Therefore, the ash content in the liquid stream was 2.21%, which indicates that the actual mineral load in the aqueous matrix was not particularly high or limiting for the enzymatic hydrolysis process.

The high ash percentage on a dry basis mainly reflects the low amount of dry matter present in the wastewater and the contribution of lime-derived calcium salts, sulphides and other inorganic compounds typical of the unhairing–liming stage. However, after concentration or drying of the hydrolysate, this mineral fraction becomes more relevant, since salts remain in the final product and may influence its conductivity, formulation and optimal application dose.

Therefore, the ash content does not prevent the valorisation of unhairing–liming wastewater, but it should be considered in downstream applications, especially for agricultural use. Appropriate dilution of the final hydrolysate is necessary to avoid excessive salinity and possible inhibitory effects during germination or early plant growth.

The elemental composition of both streams is shown in Table 2. The unhairing–liming wastewater presented notably higher concentrations of calcium, magnesium, sodium, total chromium and zinc than lime-fleshing wastewater. In particular, calcium reached 4171.00 mg/kg in unhairing–liming wastewater compared with 888.2 mg/kg in lime-fleshing wastewater, reflecting the intense use of lime during unhairing and the subsequent carry-over of calcium compounds into the effluent. Magnesium was also markedly higher in unhairing–liming wastewater, with 7367.54 mg/kg, while sodium increased from 483.4 mg/kg in lime-fleshing wastewater to 2744.34 mg/kg in unhairing–liming wastewater. These differences confirm the stronger inorganic contribution of the unhairing–liming stream.

Regarding heavy metals, none of the measured elements exceeded the regulatory limits established for their potential use in biostimulant applications, according to Regulation (EU) 2019/1009 and Royal Decree 506/2013 [25,39]. Particularly relevant is the absence of detectable Cr (VI), which remained below the limit of quantification in both streams. Total chromium was detected in unhairing–liming wastewater at 13.66 mg/kg, but this value remained compatible with the intended application and does not restrict its use under the evaluated conditions. Cadmium, lead, mercury, copper and arsenic were also below quantification limits or present at very low levels. These results support the potential use of both effluents as raw materials for the production of hydrolysates intended for agricultural applications, provided that the final products also comply with the corresponding regulatory requirements.

The pH and mineral composition of both streams also provide relevant information for process design. The alkaline pH of unhairing–liming wastewater may favour protein solubilisation, but it must be adjusted to the optimum pH range of the selected enzymes before hydrolysis. Lime-fleshing wastewater, with a pH close to neutrality, requires less correction, which may simplify process conditioning. However, its higher solid and protein contents may require adequate homogenisation to ensure efficient mass transfer and reproducible hydrolysis. Therefore, both streams present different operational advantages: unhairing–liming wastewater offers a more diluted but keratin-containing substrate, whereas lime-fleshing wastewater provides a more concentrated collagen-rich protein stream.

**Figure 2:**
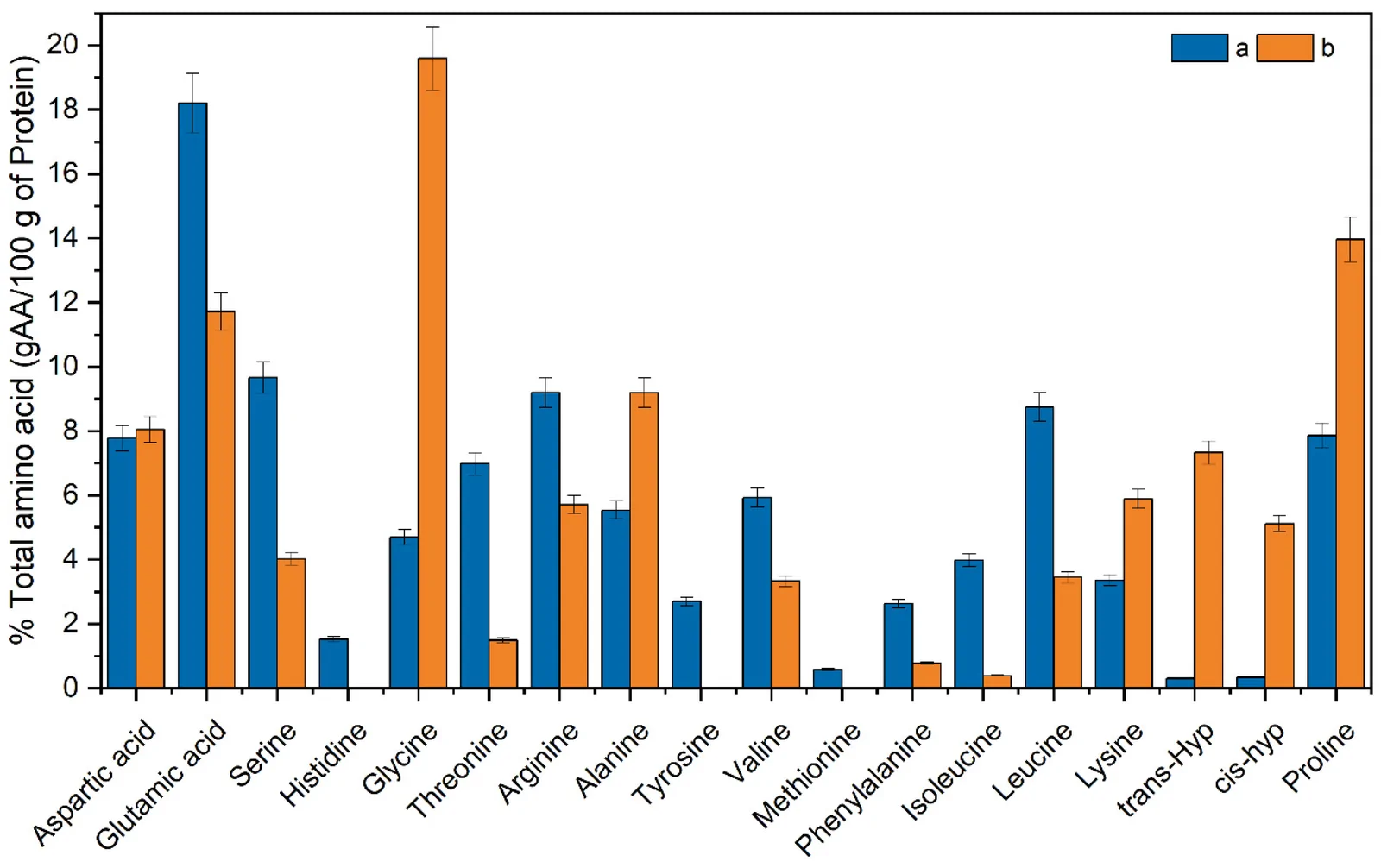
Total amino acid profile from streams used: (a) unhairing–liming wastewater and (b) fleshing–liming wastewater.

Additionally, an amino acid profile analysis of the different raw materials used in this study was performed in order to evaluate their potential efficacy in biostimulant applications. This characterisation provides insight into their nutritional composition and allows for the assessment of bioactive compounds that may contribute to plant growth promotion and stress tolerance mechanisms (Figure 2).

The amino acid profile of both raw materials was also analysed to assess their potential suitability for hydrolysed protein with a growth-stimulating effect. This characterisation is relevant because the biological activity of protein hydrolysates is strongly influenced not only by the total amino acid content, but also by the relative abundance of specific amino acids involved in plant metabolism, nutrient assimilation and stress response.

The comparison of the amino acid profiles shown in Figure 2 highlights important differences between the two raw materials, which are directly related to their origin within the beamhouse process. Unhairing–liming wastewater showed a more heterogeneous amino acid profile, with glutamic acid as the predominant amino acid, followed by serine, arginine, leucine, proline, aspartic acid, valine, alanine and glycine. This composition is consistent with the mixed nature of this stream, which contains proteinaceous material derived from hair degradation, epidermal residues, soluble non-collagenous proteins and partially solubilised collagen fragments. The relatively higher contribution of amino acids such as serine, leucine, valine and glutamic acid suggests a stronger influence of keratin-derived and non-collagenous proteins released during the alkaline unhairing process.

In contrast, lime-fleshing wastewater showed a clearly collagen-like amino acid profile, with glycine as the major amino acid, followed by proline, glutamic acid, aspartic acid, alanine, trans-hydroxyproline, lysine and arginine. The predominance of glycine, proline and hydroxyproline is characteristic of collagen-rich materials and confirms that this stream mainly originates from connective tissue removed during fleshing after liming. This difference is relevant because collagen-derived proteins are generally more accessible to proteolytic hydrolysis than keratin-rich materials, particularly after the swelling and partial solubilisation caused by the liming stage.

Therefore, the amino acid profiles confirm that the two wastewaters should not be considered equivalent substrates. Unhairing–liming wastewater represents a more complex and compositionally heterogeneous protein stream, whereas lime-fleshing wastewater constitutes a more concentrated collagen-derived raw material. These differences help explain the different enzymatic responses observed later in the study.

Several of the amino acids identified in both streams are directly related to plant growth and stress tolerance mechanisms. Glutamic and aspartic acids are involved in nitrogen metabolism and nutrient assimilation. Arginine has been associated with improved chlorophyll production and nitrogen-related pathways. Proline plays a key role in osmotic regulation and can help mitigate salinity stress. Glycine contributes to structural and metabolic processes, and leucine and serine may support plant growth and physiological activity [21,22,23,24,42]. Therefore, the amino acid composition of both wastewaters supports their potential use as precursors for amino acid-based biostimulants.

Taken together, the initial characterisation confirms that both unhairing–liming wastewater and lime-fleshing wastewater are suitable streams for sequential enzymatic hydrolysis. Lime-fleshing wastewater provides a more concentrated collagen-derived protein stream, whereas unhairing–liming wastewater offers a more diluted but compositionally different stream, enriched in the keratin-related amino acids and mineral components typical of the unhairing process. In both cases, fat content is moderate and does not appear to limit enzyme accessibility, while ash and mineral contents can be managed through pH adjustment and process conditioning. These data provide the basis for the subsequent hydrolysis experiments and support the use of tannery wastewaters as secondary raw materials for obtaining functional protein hydrolysates within an environmentally friendly waste recovery approach.

### 3.2. Selection of the Enzyme Used in the Enzymatic Hydrolysis

Three enzymes with endo-activity and one enzyme with exo-activity that work at different temperatures and pH values were selected. The working conditions at which each enzyme shows its maximum activity are shown in Table 3.

The enzymatic hydrolysis strategy was designed considering the different chemical nature of the two tannery wastewater streams evaluated in this study. Since protein accessibility, previous alkaline treatment, pH, mineral content and the degree of protein solubilisation may strongly affect enzyme performance, proteases with different catalytic behaviours and optimum working conditions were initially screened. This approach made it possible to identify the most suitable enzymatic system for each wastewater prior to further process optimisation.

The enzyme screening included two alkaline endoproteases, Alcalase (ALC) and Bioprotease (BPT), which can cleave internal peptide bonds and generate intermediate peptide fragments. In addition, NP, a proteolytic enzyme working under strongly alkaline conditions, was evaluated because of the alkaline nature of the unhairing–liming stream. Finally, Pancreatin (PAN), a neutral proteolytic enzyme with exo-activity, was used as a second hydrolysis step to promote the further breakdown of peptides and increase the release of free amino acids. Therefore, the process was structured as a sequential enzymatic treatment combining an initial proteolytic fragmentation step with a subsequent amino acid-releasing stage.

Table 4 and Table 5 summarise the results obtained after the enzymatic hydrolysis of unhairing–liming wastewater and lime-fleshing wastewater, respectively, using the different enzyme combinations under their optimal working conditions.

In the case of unhairing–liming wastewater, the results showed that alkaline endoproteases were generally more effective than the enzyme operating under strongly alkaline conditions. Among the tested conditions, the best performance was obtained with the sequential treatment based on 1.5% ALC followed by 1% PAN, which provided the highest yield (70.76%), the highest protein recovery (80.87%) and the highest free amino acid content (11.42%). This result indicates that Alcalase was able to efficiently hydrolyse the proteinaceous material present in this wastewater, generating peptides that were subsequently further degraded by Pancreatin into free amino acids.

The use of 2% ALC also led to a high protein content in the final hydrolysate (34.96%) and a protein recovery of 67.86%. However, increasing the ALC dosage from 1.5% to 2% did not improve the process. On the contrary, yield and free amino acid release decreased, suggesting that excessive enzyme loading did not provide additional hydrolytic benefit under the evaluated conditions. This behaviour may be related to enzyme saturation, reduced substrate accessibility or the formation of peptides that were not further converted into free amino acids during the second hydrolysis stage.

BPT showed lower efficiency than ALC for this wastewater. Protein recoveries ranged from 48.36% to 54.93%, while yields remained between 46.29% and 52.37%. Although increasing BPT loading from 1.5% to 2% improved yield, protein content and free amino acid release, the values remained below those obtained with Alcalase. This suggests that BPT was less suited to the specific protein matrix present in unhairing–liming wastewater, probably because this stream contains a heterogeneous mixture of keratin-derived fragments, collagen-derived peptides, sulphide-treated proteins and suspended solids that require a more efficient endoproteolytic cleavage.

Enzymatic hydrolysis with NP produced intermediate results, with yields between 55.33% and 63.32% and protein recoveries between 58.67% and 65.43%. Nevertheless, the free amino acid contents remained around 7–8%, lower than the best ALC/PAN hydrolysis. Although NP operates under highly alkaline conditions, which could initially appear favourable for unhairing–liming wastewater, the results suggest that this enzyme did not provide sufficient specificity or efficiency to maximise amino acid release from the proteinaceous material present in this effluent.

Collectively, these results demonstrate that the combination of ALC and PAN was the most suitable enzymatic system for unhairing–liming wastewater. ALC provided efficient initial peptide bond cleavage, whereas PAN contributed to the release of free amino acids during the second step. Therefore, this enzyme combination was selected for the subsequent optimisation of enzyme loading and hydrolysis conditions.

In the case of lime-fleshing wastewater, lower enzyme loadings were tested during the selection of the enzymatic system, ranging from 1.0% to 1.5% for the first hydrolysis step. This decision was based on the nature of the substrate. Unlike unhairing–liming wastewater, lime-fleshing wastewater contains proteinaceous material that has already undergone partial alkaline hydrolysis during the liming stage. As a result, part of the collagen-rich protein fraction is already solubilised in the aqueous phase, and the main objective of the enzymatic hydrolysis is not to extensively disrupt the original tissue structure, but to further reduce the molecular weight of the soluble protein fragments and increase the concentration of free amino acids suitable for biostimulant applications.

The hydrolysis of lime-fleshing wastewater was more efficient than that of unhairing–liming wastewater for all the enzymatic systems evaluated. Yields ranged from 80.66% to 94.06%, and the protein contents in the hydrolysates remained between 50.67% and 58.51% on a dry matter basis. These higher values are consistent with the higher protein concentration and greater accessibility of the collagen-derived fraction present in lime-fleshing wastewater.

The best results were obtained with 1.0% ALC followed by 1% PAN. This condition provided the highest yield (94.06%), the highest protein content (58.51%), the highest free amino acid content (16.28%) and the highest protein recovery (74.68%). Interestingly, increasing the ALC loading to 1.5% reduced the yield, free amino acid content and protein recovery. This suggests that, for lime-fleshing wastewater, the partially hydrolysed nature of the substrate makes a lower Alcalase dosage sufficient to achieve efficient protein conversion. Higher enzyme concentrations did not improve hydrolysis and may have promoted non-productive interactions or an imbalance between the first and second hydrolysis steps.

BPT also provided satisfactory free amino acid contents, particularly at 1.0% enzyme loading, where 15.13% free amino acids were obtained. However, the protein recovery values achieved with BPT were clearly lower than those obtained with ALC, remaining between 56.45% and 58.28%. This indicates that, although BPT can promote amino acid release, its overall ability to recover and solubilise the protein fraction was lower than that of Alcalase under the conditions tested.

Enzymatic hydrolysis with NP showed the lowest protein recovery values, ranging from 50.19% to 52.23%, and free amino acid contents between 11.88% and 12.66%. These results confirm that, although NP can hydrolyse part of the proteinaceous fraction, it is less effective than ALC and BPT for producing amino acid-rich hydrolysates from lime-fleshing wastewater. This may be due to the collagen-rich nature of this stream, which appears to be more efficiently hydrolysed by alkaline endoproteases such as Alcalase.

The comparison between both wastewater streams reveals clear differences in enzymatic behaviour. Unhairing–liming wastewater required higher enzyme loads and produced lower yields and free amino acid contents, probably due to its more complex composition, lower protein concentration, higher mineral content and the presence of keratin-derived material. In contrast, lime-fleshing wastewater responded more efficiently to enzymatic hydrolysis, requiring lower enzyme loading and generating hydrolysates with higher yield, protein content and free amino acid concentration. This confirms that the origin and previous treatment history of the wastewater strongly determine hydrolysis performance.

Based on these results, Alcalase was selected as the most effective endo-acting enzyme for both tannery wastewater streams, while Pancreatin was maintained as the exo-acting enzyme for the second hydrolysis step. The ALC/PAN sequential system was, therefore, selected for process optimisation, as it provided the best balance between hydrolysate yield, protein recovery and free amino acid release in both unhairing–liming wastewater and lime-fleshing wastewater.

### 3.3. Optimisation of Enzyme Loading in the Sequential ALC/PAN Hydrolysis Process

After identifying Alcalase (ALC) as the most efficient endo-acting enzyme and Pancreatin (PAN) as the most suitable exo-acting enzyme for the second hydrolysis step, the next stage of the study focused on optimising the enzyme loading for each wastewater stream. This optimisation was necessary because unhairing–liming wastewater and lime-fleshing wastewater differ markedly in protein concentration, degree of previous alkaline hydrolysis, mineral composition, solid content and protein accessibility. These differences may affect the amount of enzyme required to achieve an efficient conversion of the proteinaceous fraction into soluble peptides and free amino acids.

The optimisation was carried out by varying the ALC concentration while maintaining the PAN loading constant at 1.0%. For unhairing–liming wastewater, ALC was evaluated in the range of 0.5–2.0%, whereas for lime-fleshing wastewater, a lower range of 0.5–1.5% was selected. This difference in the tested enzyme range was based on the nature of the two streams. Lime-fleshing wastewater contains proteinaceous matter that has already undergone partial alkaline hydrolysis during the liming stage and is, therefore, expected to be more accessible to enzymatic cleavage. In contrast, unhairing–liming wastewater is a more diluted and compositionally complex stream, containing keratin-derived compounds, collagen fragments, suspended solids and a higher mineral fraction, which may require higher enzyme loading to achieve efficient hydrolysis.

The pH, temperature and reaction time were maintained according to the optimal conditions defined in the experimental section. The selection of the optimal hydrolysis condition was mainly based on the maximum free amino acid content obtained in the dried hydrolysate, since this parameter is highly relevant for the intended application as an amino acid-based plant biostimulant. Nevertheless, hydrolysis yield, total amino acid content and protein recovery were also considered. Yield reflects the amount of hydrolysed product obtained from the treated wastewater, whereas protein recovery provides information on the efficiency with which the initial proteinaceous fraction is converted into the final hydrolysate.

The results obtained with a variation of enzyme load are shown in Table 6 and Table 7.

The results obtained for unhairing–liming wastewater show that enzyme loading had a clear influence on the hydrolysis response. At low ALC concentration (0.5%), the process yielded 58.12% hydrolysate and 5.90% free amino acids, indicating that the enzyme amount was insufficient to maximise peptide bond cleavage and amino acid release. When the ALC loading was increased to 1.0%, the yield rose markedly to 81.50%, and protein recovery reached the highest value obtained for this wastewater, 91.10%. This suggests that 1.0% ALC was highly effective in solubilising and recovering the protein fraction present in the wastewater.

Nevertheless, the highest free amino acid content was not obtained under the condition with the maximum yield or protein recovery. The free amino acid content increased progressively from 5.90% at 0.5% ALC to 11.42% at 1.5% ALC. This indicates that, although 1.0% ALC promoted higher protein recovery, increasing the enzyme loading to 1.5% favoured deeper hydrolysis and enhanced the conversion of peptides into free amino acids during the sequential ALC/PAN treatment. This distinction is important because, for biostimulant applications, the concentration of free amino acids is a key parameter, as these compounds are directly involved in plant metabolism, nutrient assimilation and stress response.

At ALC loadings above 1.5%, the hydrolysis performance decreased, with lower yield, protein recovery and free amino acid content. Since no kinetic study was performed, this behaviour should not be interpreted as direct evidence of a specific kinetic limitation. Instead, the results indicate that increasing the enzyme loading beyond the optimal value did not improve the final hydrolysis outcome under the selected process conditions.

In this study, the extent of hydrolysis was assessed through the final composition of the recovered hydrolysates, particularly hydrolysate yield, protein recovery, total amino acid content and free amino acid content. These parameters reflect the amount of proteinaceous material solubilised and converted into amino acid-containing fractions during the process. Therefore, the decrease observed at higher ALC loadings indicates that excessive enzyme addition did not lead to a higher recovery of soluble protein or a higher release of free amino acids. This behaviour may be related to substrate accessibility limitations, accumulation of less hydrolysable peptide fractions, product effects or non-productive enzyme–substrate interactions.

Based on these results, the selection of the optimal condition was not made only according to the highest protein recovery but also according to the intended functionality of the final product. Although the treatment with 1.0% ALC provided the highest protein recovery, 91.1%, the condition selected as optimal for unhairing–liming wastewater was 1.5% ALC/1.0% PAN because it produced the highest free amino acid content, 11.4%. This criterion was considered more appropriate for the objective of the study, which was to obtain amino acid-based protein hydrolysates with potential application as plant biostimulants.

Protein recovery remains an important parameter because it reflects the overall efficiency of converting the initial proteinaceous fraction into the final hydrolysate. However, this parameter includes both amino acids present in peptides or protein fragments and amino acids present in free form. Therefore, a higher protein recovery does not necessarily indicate a higher concentration of immediately bioavailable amino acids. In the case of the 1.0% ALC treatment, the high protein recovery suggests efficient solubilisation of the protein fraction, but the free amino acid content remained lower than that obtained with 1.5% ALC.

For potential biostimulant applications, free amino acids are considered one of the most relevant bioactive fractions because they can be directly absorbed or rapidly used by plants in metabolic processes related to nitrogen assimilation, chlorophyll synthesis, nutrient uptake and stress response. Peptide-bound amino acids and larger protein fragments may also contribute to plant growth promotion, but their effect is generally less direct because they require further degradation or transformation before becoming fully available to the plant.

Therefore, the 1.5% ALC/1.0% PAN condition was selected because it provided the best functional balance for the target application: a high hydrolysate yield, adequate protein recovery and the highest concentration of free amino acids. This approach prioritises the generation of a hydrolysate with greater potential bioavailability and suitability for agricultural use, rather than maximising protein recovery alone.

**Figure 3:**
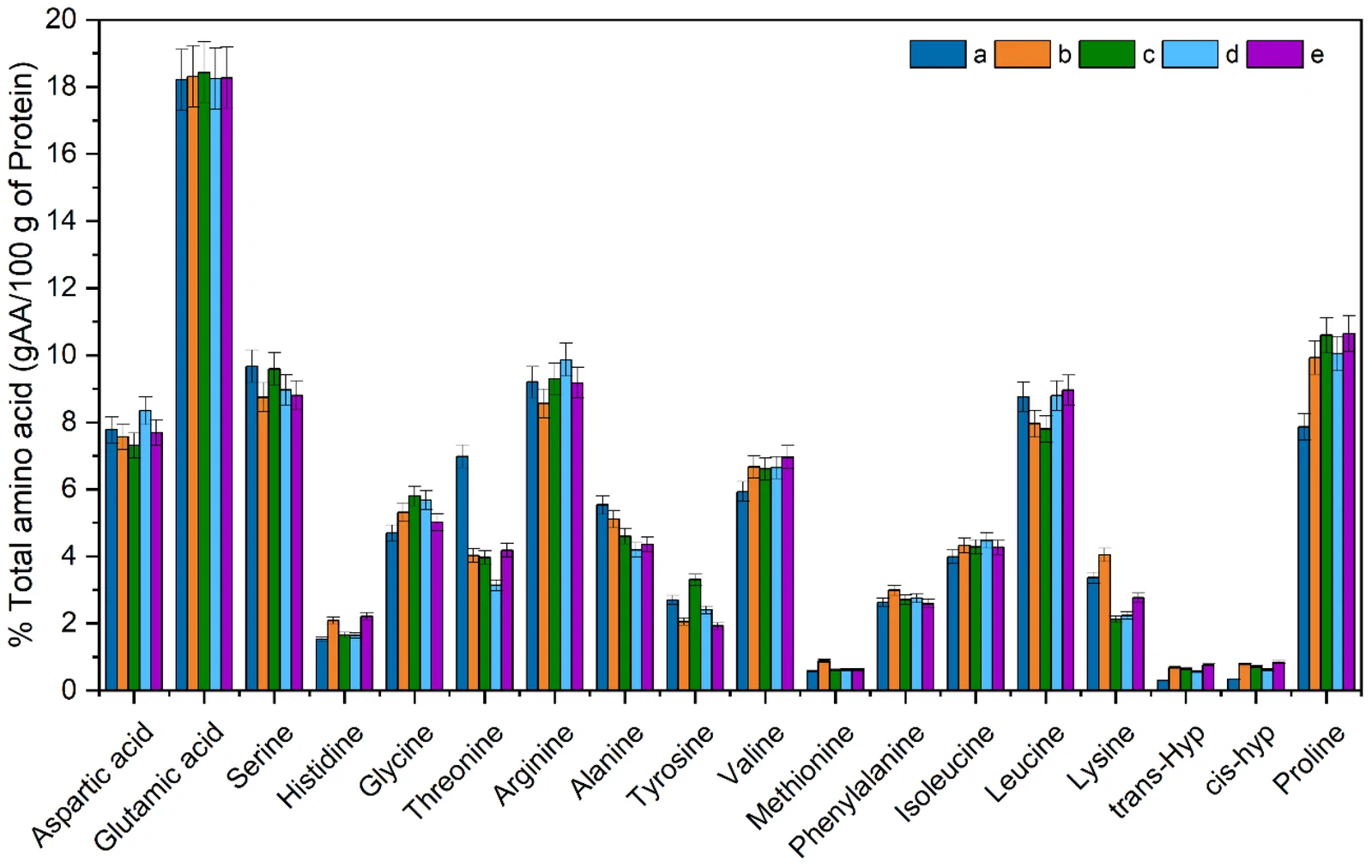
Comparison of the total amino acid profile of the (a) unhairing–liming wastewater, different hydrolysed proteins obtained from unhairing–liming wastewater (b) 1.0% ALC, 1% PAN, (c) 1.25% ALC, 1% PAN, (d) 1.5% ALC, 1% PAN and (e) 1.75% ALC, 1% PAN.

After selecting the optimal enzymatic condition, the amino acid profiles were analysed to further evaluate the potential of the hydrolysates as hydrolysed protein with a growth-stimulating effect. Figure 3 compares the total amino acid profile of the original unhairing–liming wastewater with the hydrolysates obtained under the different ALC/PAN conditions. This comparison provides information on whether the sequential enzymatic treatment modified the amino acid distribution or preserved the characteristic profile of the original proteinaceous stream.

The amino acid distribution of the hydrolysates generally followed the same trend observed in the original unhairing–liming wastewater, confirming that the enzymatic process mainly depolymerised the proteinaceous fraction without causing major changes in the relative amino acid composition. Glutamic acid remained the predominant amino acid, followed by proline, arginine, serine, leucine, aspartic acid, valine and glycine. The hydrolysates showed relevant proportions of amino acids associated with plant physiological processes, including aspartic acid (7.6%), arginine (9.3%), serine (8.7%), leucine (8.0%), valine (6.6%) and glycine (5.3%). These results support the potential of the hydrolysates obtained from unhairing–liming wastewater as amino acid-based biostimulant ingredients.

The relative decrease in proline content observed after enzymatic hydrolysis should not be interpreted as a specific selectivity of Alcalase towards proline residues. Alcalase 2.4 L is mainly based on subtilisin A, an alkaline serine endopeptidase with broad substrate specificity that catalyses the hydrolysis of internal peptide bonds in proteins and peptides. However, proline-containing sequences may show a different hydrolysis behaviour due to the particular cyclic structure of proline, which imposes conformational restrictions on the peptide backbone and can reduce the accessibility of neighbouring peptide bonds to proteases.

This effect is especially relevant in collagen-derived substrates, where proline and hydroxyproline are abundant and contribute to the stability of the collagen structure. Previous studies have shown that proline residues can markedly influence the susceptibility of adjacent peptide bonds to proteolytic cleavage, and that the presence of proline in specific positions may strongly reduce or even prevent hydrolysis by subtilisin-like serine proteases. Therefore, the lower relative proline content detected in the soluble hydrolysate fraction may be associated with the lower release of proline-rich sequences into a solution or their preferential retention in higher-molecular-weight or non-hydrolysed fractions removed during centrifugation/filtration [43,44].

The maximum ALC loading in lime-fleshing wastewater (Table 7) showed a different trend from that observed for unhairing–liming wastewater (Table 6). In this case, the best hydrolysis performance was obtained at lower enzyme loading. Increasing the ALC from 0.5% to 0.7% produced a marked improvement in all the key parameters evaluated. The yield increased from 72.03% to 98.42%, free amino acid content rose from 12.46% to 16.97%, and protein recovery increased from 52.66% to 92.19%. These results indicate that 0.7% ALC was sufficient to efficiently hydrolyse the protein fraction present in lime-fleshing wastewater.

The higher sensitivity of lime-fleshing wastewater to lower enzyme dosages can be explained by the previous alkaline treatment undergone by the stream during liming. This treatment partially hydrolyses and solubilises collagen-rich proteins, increasing their accessibility to proteolytic enzymes. Therefore, the enzymatic process mainly needs to further fragment already solubilised protein chains and promote the release of free amino acids, rather than disrupt a highly resistant or poorly accessible protein matrix.

Although 1.0% ALC led to the highest total amino acid content, 58.51%, its yield, protein recovery and free amino acid content were slightly lower than those obtained at 0.7% ALC. This indicates that a higher total amino acid content does not necessarily correspond to the best hydrolysis condition when the objective is to maximise amino acid release and process efficiency. From a biostimulant production perspective, the 0.7% ALC/1.0% PAN condition provided the best balance between yield, protein recovery and free amino acid generation.

Further increases in ALC loading, from 1.25% to 1.5%, reduced yield, free amino acid content and protein recovery. As observed for unhairing–liming wastewater, this decrease suggests that excessive enzyme loading does not improve hydrolysis once the accessible protein fraction has been converted. At high enzyme/substrate ratios, the reaction may become limited by the availability of hydrolysable peptide bonds rather than by enzyme concentration. Consequently, the addition of more enzyme does not provide further conversion and may reduce the efficiency of the process from both technical and economic perspectives.

Therefore, the maximum ALC loading for lime-fleshing wastewater was established at 0.7% in combination with 1.0% PAN. This condition yielded 98.42% hydrolysate, 16.97% free amino acids and 92.19% protein recovery, demonstrating that lime-fleshing wastewater is more readily hydrolysed than unhairing–liming wastewater and requires a lower endoprotease dosage to obtain an amino acid-rich product.

The comparison between the two wastewater streams confirms that substrate composition and previous processing history strongly determine enzyme requirements. Unhairing–liming wastewater required a higher ALC loading to maximise free amino acid release, probably due to its lower protein concentration, higher mineral content and the presence of keratin-derived components. In contrast, lime-fleshing wastewater behaved as a more accessible collagen-rich stream, allowing efficient hydrolysis at a lower ALC concentration. These differences highlight the importance of adapting enzymatic conditions to each wastewater stream rather than applying a single generic hydrolysis protocol.

**Figure 4:**
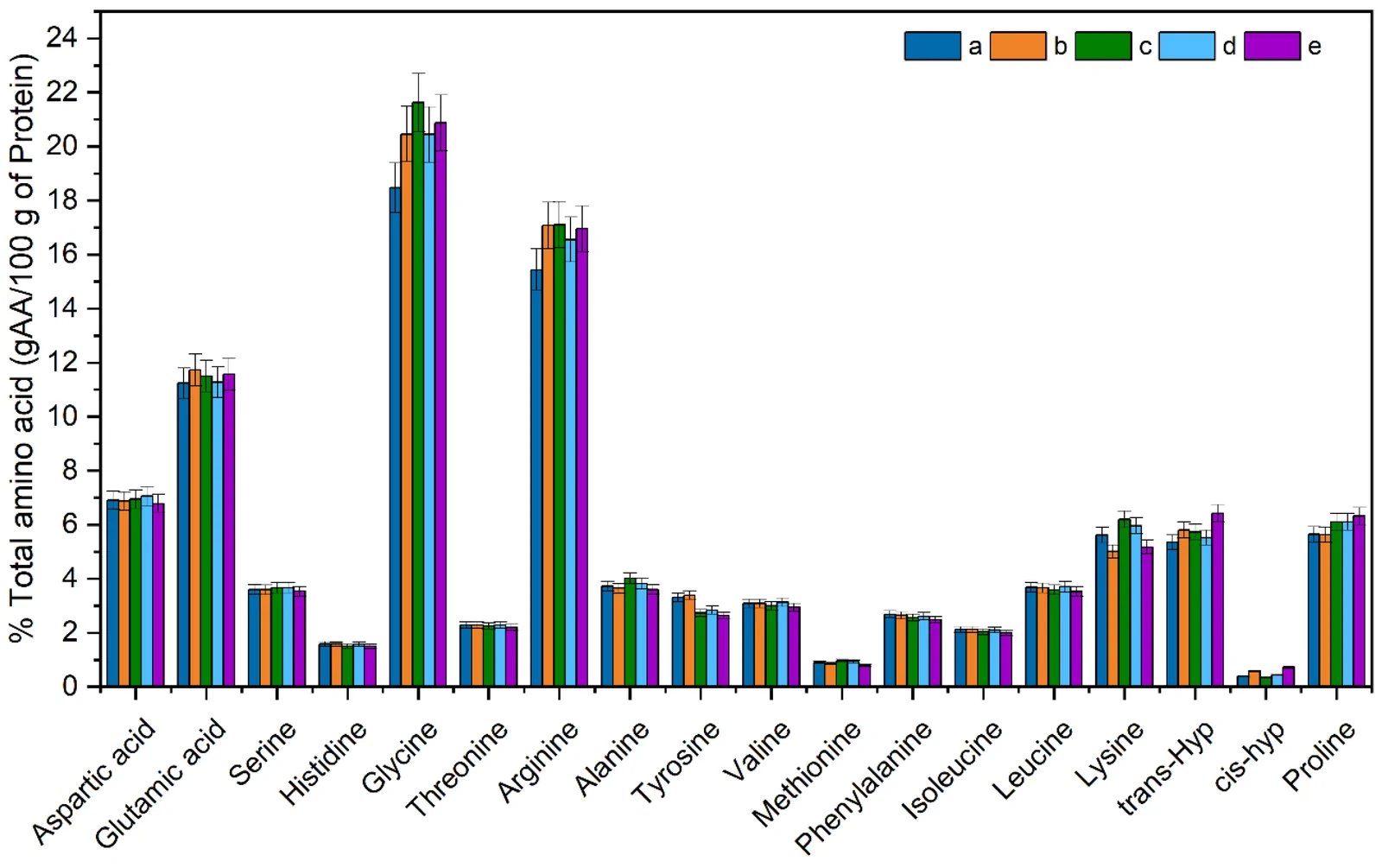
Comparison of the total amino acid profile of the different hydrolysed proteins obtained from lime-fleshing wastewater: (a) 0.5% ALC, 1% PAN, (b) 0.75% ALC, 1% PAN, (c) 1.0% ALC, 1% PAN, (d) 1.25% ALC, 1% PAN and (e) 1.5% ALC, 1% PAN.

Figure 4 shows the total amino acid profiles of the protein hydrolysates obtained from lime-fleshing wastewater under the different enzymatic hydrolysis conditions. This analysis was carried out to determine whether the enzyme loading influenced the amino acid distribution of the resulting hydrolysates and to assess their suitability as amino acid-based biostimulant ingredients.

In general, the amino acid distribution of the hydrolysates followed the same pattern as that observed in the original lime-fleshing wastewater, indicating that the sequential enzymatic process mainly promoted the depolymerisation of the proteinaceous fraction without causing substantial changes in the amino acid profile. Glycine remained the predominant amino acid, followed by arginine, glutamic acid, aspartic acid, proline, trans-hydroxyproline and lysine. The hydrolysates contained relevant proportions of amino acids associated with plant physiological processes, including glycine (20.8%), arginine (15.9%), glutamic acid (11.5%), aspartic acid (6.8%), proline (6.3%), trans-hydroxyproline (5.8%) and lysine (5.7%). These results confirm that lime-fleshing wastewater can be converted into hydrolysates with an amino acid composition of interest for potential biostimulant applications.

A notable difference between the original lime-fleshing wastewater and the corresponding hydrolysates was the relative decrease in proline content after enzymatic treatment [30]. This reduction may be explained by the specific behaviour of collagen-derived sequences during hydrolysis. Lime-fleshing wastewater is mainly composed of collagen-rich protein fragments, in which glycine, proline and hydroxyproline are characteristic amino acids. However, proline-containing peptide bonds are known to be more resistant to enzymatic cleavage because the cyclic structure of proline imposes conformational restrictions on the peptide backbone [30]. As a result, during hydrolysis, proline may remain preferentially associated with less soluble, higher molecular weight peptide fractions or with residual non-hydrolysed material removed after centrifugation or filtration. Consequently, the soluble hydrolysate fraction may show a lower relative proline proportion compared to the initial wastewater, while other amino acids more readily released into solution become proportionally enriched.

The differences observed between the hydrolysates obtained from lime-fleshing wastewater and those previously obtained from unhairing–liming wastewater also reflect the different origin of the proteinaceous material in both streams. In unhairing–liming wastewater, glutamic acid was the predominant amino acid, followed by proline, which is consistent with a more heterogeneous protein composition including keratin-derived material from hair degradation together with collagen fragments. In contrast, lime-fleshing wastewater showed a clearly collagen-like profile, with glycine as the major amino acid and arginine as the second most abundant amino acid in the hydrolysates.

This distinction is relevant because amino acid composition can strongly influence the growth-stimulating effect of the final product. Glycine, arginine, glutamic acid, aspartic acid, proline and lysine are involved in different plant metabolic pathways related to nitrogen assimilation, chlorophyll synthesis, osmotic regulation, nutrient uptake and stress tolerance. Therefore, the different amino acid profiles obtained from unhairing–liming wastewater and lime-fleshing wastewater suggest that each hydrolysate may exert complementary effects on plant development, depending on the relative abundance of specific amino acids released during enzymatic hydrolysis.

**Figure 5:**
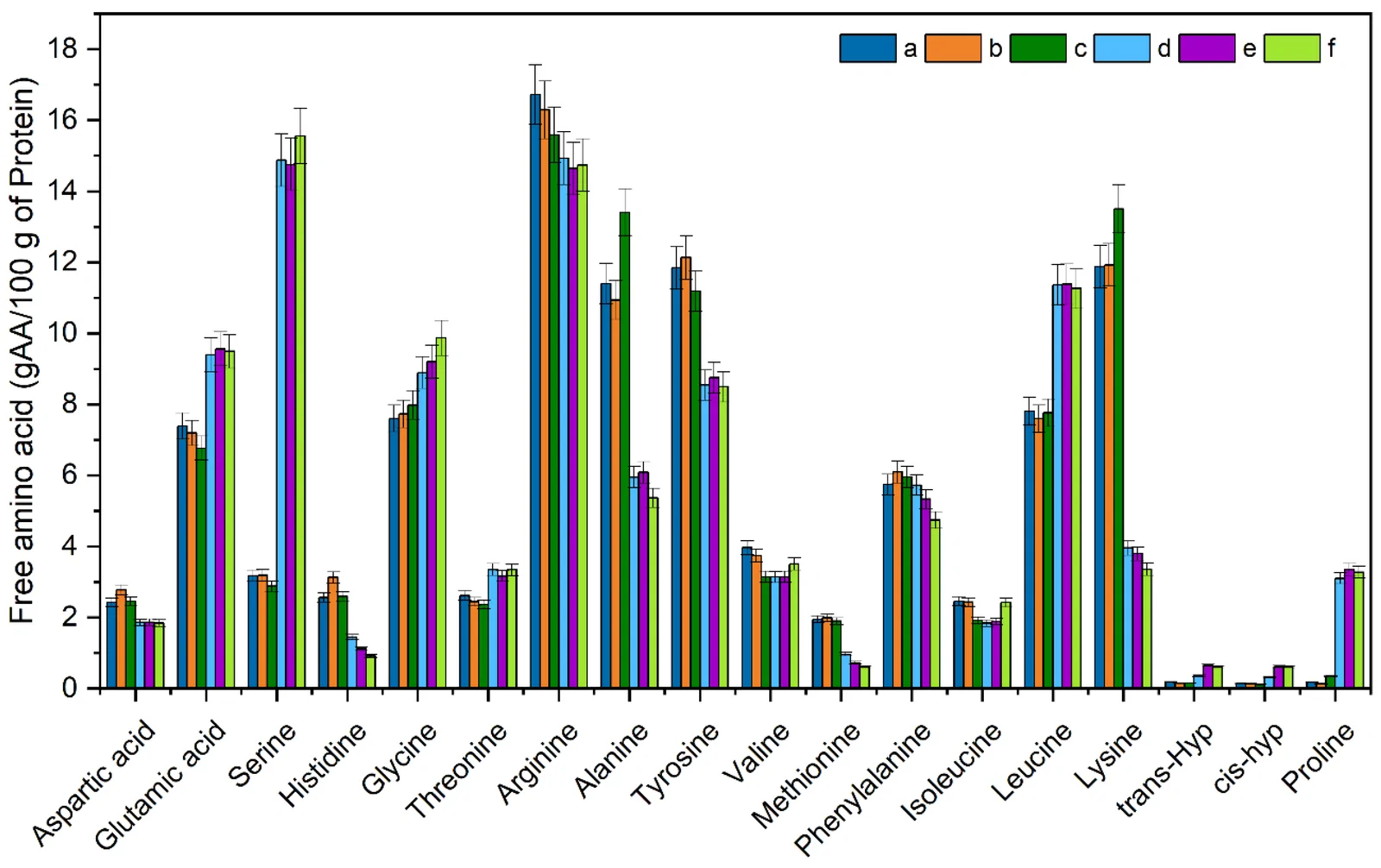
Comparison of the free amino acid profile of the different hydrolysed protein obtained from lime-fleshing wastewater, (a) 1.0% ALC, 1% PAN, (b) 1.5% ALC, 1% PAN, (c) 1.25% ALC, 1% PAN, and different hydrolysed protein obtained from unhairing–liming wastewater (d) 0.75% ALC, 1% PAN, (e) 1.0% ALC, 1% PAN and (f) 1.25% ALC, 1% PAN.

The following Figure 5 shows the comparative free amino acid profile of the hydrolysates obtained from the evaluated unhairing–liming wastewater.

The free amino acid composition of the protein hydrolysates obtained from the two tannery wastewater streams showed marked differences, indicating that the origin and composition of the initial proteinaceous substrate strongly influenced the final hydrolysate profile. Arginine was the predominant free amino acid in both hydrolysates, although its relative abundance was higher in the hydrolysate produced from lime-fleshing wastewater (16.3%) than in that obtained from unhairing–liming wastewater (14.6%). This high arginine content is particularly relevant for potential biostimulant applications, since arginine is involved in nitrogen metabolism and can act as a precursor of polyamines and nitric oxide-related pathways, which are associated with plant growth regulation, stress responses and chlorophyll biosynthesis.

The hydrolysates obtained from unhairing–liming wastewater showed significantly higher levels of serine, glutamic acid, leucine and glycine. This difference may be related to the contribution of keratin-derived proteins released during the unhairing process, together with partially solubilised collagen fragments. From an agronomic perspective, the enrichment in these amino acids is relevant because glutamic acid is directly involved in nitrogen assimilation and amino acid biosynthesis, serine participates in photorespiration and one-carbon metabolism, leucine may contribute to protein synthesis and metabolic regulation, and glycine is involved in chlorophyll formation and stress-related physiological processes. Therefore, the higher abundance of these amino acids may confer a specific functional profile to the unhairing–liming wastewater hydrolysate.

In contrast, the hydrolysates obtained from lime-fleshing wastewater presented significantly higher proportions of lysine, tyrosine, alanine, aspartic acid and histidine. This composition is consistent with the collagen-rich nature of lime-fleshing residues, which undergo partial alkaline solubilisation during the liming stage and are subsequently further depolymerised during enzymatic hydrolysis. Aspartic acid and alanine are closely linked to nitrogen and carbon metabolism, while lysine and histidine may contribute to plant nutritional balance and stress-related responses. Tyrosine is also of interest because it can participate in the biosynthesis of phenolic compounds and secondary metabolites involved in plant defence mechanisms.

These differences in the free amino acid profiles suggest that the two hydrolysates should not be considered equivalent products, even though both are obtained from tannery wastewater streams and both contain high levels of bioavailable amino acids. The lime-fleshing wastewater hydrolysate appears to be more enriched in the amino acids associated with collagen-derived protein fractions, whereas the unhairing–liming wastewater hydrolysate shows a profile more influenced by keratin-related and mixed proteinaceous material. Since the growth-stimulating effect of protein hydrolysates depends not only on the total free amino acid content but also on the relative abundance of specific amino acids, these compositional differences may lead to different effects on plant growth, nutrient uptake, chlorophyll production and tolerance to abiotic stress [21,22,23,24,42].

Overall, the results demonstrate that the enzymatic valorisation of both wastewater streams can generate amino acid-rich hydrolysates with differentiated functional profiles. This supports the relevance of comparing both streams, as their distinct amino acid compositions may provide complementary growth-stimulating effects depending on the crop, dose and target physiological response.

These observations align with the ANOVA results, which also indicated significant differences (*p* < 0.001) among amino acids, confirming that the compositional variability between samples is statistically supported rather than experimental noise.

Finally, Table 8 presents the full physicochemical characterisation of the protein hydrolysates obtained under the selected optimal enzymatic conditions for each tannery wastewater stream. This characterisation was carried out to evaluate their suitability as free amino acid-based biostimulants and to verify their compliance with the main compositional parameters required for this type of product, including total nitrogen, organic nitrogen, ammonia nitrogen, total amino acids, free amino acids, dry matter, organic matter, ash content, density and pH.

The two hydrolysates showed a composition consistent with protein-derived potential biostimulant products, although clear differences were observed depending on the wastewater used as raw material. The hydrolysate obtained from lime-fleshing wastewater presented a higher total nitrogen content, with 7.29%, compared with 6.12% for the hydrolysate produced from unhairing–liming wastewater. This difference is coherent with the higher proteinaceous load of lime-fleshing wastewater and confirms that this stream provides a more concentrated collagen-derived substrate for enzymatic hydrolysis.

Despite this difference in total nitrogen, both hydrolysates showed very similar organic nitrogen contents, with values of 5.22% for the unhairing–liming wastewater hydrolysate and 5.13% for the lime-fleshing wastewater hydrolysate. This indicates that, in both cases, most of the nitrogen present in the final product is associated with organic compounds, mainly peptides and amino acids, rather than inorganic nitrogen forms.

In this context, the organic nitrogen fraction should be understood as the nitrogen contained in soluble proteinaceous compounds generated during enzymatic hydrolysis, rather than as a single chemical species. In protein hydrolysates, this fraction is mainly associated with free amino acids, short peptides, oligopeptides and soluble peptide/protein fragments. These compounds are considered relevant for potential biostimulant applications because protein hydrolysates are generally composed of amino acids, peptides and polypeptides, which may contribute to plant nutrient assimilation, photosynthetic activity and stress response.

Specific examples of bioactive organic nitrogen compounds that may be present in the hydrolysates include free amino acids such as glycine, proline, hydroxyproline, glutamic acid, aspartic acid, alanine, serine, arginine and lysine, as well as low molecular weight peptides containing these amino acid residues. In particular, glutamic acid and aspartic acid are related to nitrogen metabolism, arginine and lysine are nitrogen-rich amino acids, and proline/hydroxyproline-containing compounds may be relevant in stress-related responses and are characteristic of collagen-derived substrates. Therefore, the organic nitrogen measured in the hydrolysates is mainly associated with amino acid- and peptide-based compounds, with potential functional relevance for hydrolysed protein with growth-stimulating activity.

The ammonia nitrogen content remained low in both hydrolysates, with values of 0.52% and 0.56% for the unhairing–liming and lime-fleshing wastewater hydrolysates, respectively. These moderate values suggest that the enzymatic process did not promote excessive deamination of amino acids or uncontrolled protein degradation. This is important because high ammonia nitrogen levels may indicate the over-hydrolysis or degradation of amino acids, which could reduce the functional quality of the hydrolysate and limit its use in agricultural applications. Similarly, urea nitrogen was practically negligible in both products, with values below 0.01%, confirming that nitrogen was mainly present in organic protein-derived forms.

The most relevant differences between the two hydrolysates were observed in the total and free amino acid contents. The hydrolysate obtained from lime-fleshing wastewater showed substantially higher total amino acid content, with 52.39% on a dry matter basis, compared with 34.82% for the unhairing–liming wastewater hydrolysate. The same trend was observed for free amino acids, with 16.97% for the lime-fleshing wastewater hydrolysate and 11.42% for the unhairing–liming wastewater hydrolysate. These results confirm that lime-fleshing wastewater was more efficiently converted into an amino acid-rich hydrolysate, probably due to its higher initial protein content and to the partial solubilisation of collagen during the previous liming stage. This pre-hydrolysed condition may have increased enzyme accessibility and favoured the release of low molecular weight peptides and free amino acids during the sequential ALC/PAN hydrolysis.

In contrast, the lower amino acid content of the unhairing–liming wastewater hydrolysate can be attributed to the more diluted nature of this stream and to its more heterogeneous composition, which includes keratin-derived fragments, collagen residues, suspended solids and a higher inorganic fraction. Keratin-rich materials are generally more resistant to hydrolysis due to their compact structure and stabilising disulphide bonds. Although the alkaline unhairing stage partially disrupts this structure, the resulting proteinaceous material may still be less accessible to enzymatic attack than the collagen-rich fraction present in lime-fleshing wastewater. Therefore, the lower free amino acid content observed in the unhairing–liming hydrolysate is consistent with the different origin and chemical nature of the substrate.

Dry matter and organic matter were also higher in the hydrolysate obtained from lime-fleshing wastewater, with values of 67.86% and 64.36%, respectively, compared with 48.52% and 43.72% for the unhairing–liming wastewater hydrolysate. These results indicate that lime-fleshing wastewater generated a more concentrated final product, with a higher proportion of recoverable organic compounds. This characteristic is advantageous from a product formulation perspective, as it may reduce the amount of water in the final hydrolysate and facilitate storage, handling and application.

A relevant difference was also observed in the ash content. The hydrolysate obtained from unhairing–liming wastewater showed a markedly higher ash content, 38.27% on a dry matter basis, whereas the lime-fleshing wastewater hydrolysate presented 24.56%. This higher mineral fraction is consistent with the origin of the unhairing–liming stream, which contains lime-derived calcium salts, sulphides and other inorganic compounds used during beamhouse processing. Although the ash content is higher in this hydrolysate, it should be interpreted together with the final application dose. Excessive mineral content may limit the maximum applicable concentration due to potential salinity effects. However, at appropriate dilution rates, the presence of mineral nutrients may also contribute to the agronomic value of the product. In any case, this parameter should be carefully considered during the formulation and application of the final potential biostimulant.

The pH values of both hydrolysates were slightly acidic and very similar, with 5.82 for the unhairing–liming wastewater hydrolysate and 5.77 for the lime-fleshing wastewater hydrolysate. These values are suitable for potential biostimulant formulations and indicate that the neutralisation and enzymatic hydrolysis process successfully adjusted the initial alkaline character of the wastewater streams. A mildly acidic pH can also improve product stability and compatibility with agricultural application systems. The densities of both hydrolysates were also comparable, with values of 1.16 kg/L and 1.22 kg/L, respectively, reflecting their concentrated organic and mineral composition.

Taken together, the results shown in Table 8 demonstrate that both tannery wastewater streams can be successfully transformed into protein hydrolysates with compositional characteristics suitable for use as free amino acid-based biostimulants. The hydrolysate obtained from lime-fleshing wastewater showed the highest total nitrogen, dry matter, organic matter, total amino acid and free amino acid contents, making it the most concentrated and amino acid-rich product. In contrast, the hydrolysate derived from unhairing–liming wastewater contained lower amino acid levels but presented a differentiated composition and a higher mineral fraction, which may lead to a different agronomic response.

These differences confirm that the two hydrolysates should be considered as distinct hydrolysed proteins with growth-stimulating effects rather than equivalent formulations. Lime-fleshing wastewater appears to be a more efficient substrate for producing collagen-derived amino acid hydrolysates, while unhairing–liming wastewater provides a more complex protein–mineral matrix influenced by keratin and beamhouse chemicals. Since the growth-stimulating effect of protein hydrolysates depends not only on the total amino acid concentration but also on the balance between free amino acids, organic nitrogen, mineral content and pH, both products may offer complementary functionalities in plant growth stimulation.

Although no additional statistical analysis was applied to the parameters presented in Table 8, the results are consistent with the previous characterisation of the raw wastewaters, the enzymatic hydrolysis yields and the amino acid profiles obtained. This internal coherence supports the reliability of the dataset and confirms that the selected enzymatic hydrolysis conditions are suitable for obtaining value-added hydrolysates from tannery wastewater streams within an environmentally friendly waste recovery approach.

### 3.4. Validation of Unhairing–Liming and Lime-Fleshing Wastewater Hydrolysate as Potential Biostimulants

Potential biostimulants based on free amino acids can improve crop performance by enhancing nutrient assimilation, supporting plant metabolism and increasing tolerance to abiotic stress conditions [21,22,23,24]. Their positive effect on crop quality and yield has been previously reported, and their mode of action has been associated, among other mechanisms, with a simplification of the nitrogen cycle in plants [21,23]. The presence of free amino acids may also contribute to plant resilience under adverse environmental conditions, including high temperature, water deficit and salinity stress [22,23,24]. Nevertheless, the response of plants to protein hydrolysates depends on several factors, such as crop species, developmental stage, environmental conditions, soil characteristics, application method, hydrolysate composition and possible interactions with other agricultural inputs [45]. Therefore, determining the appropriate application dose is essential to obtain a positive physiological response and to avoid inhibitory effects caused by excessive concentrations [46].

**Figure 6:**
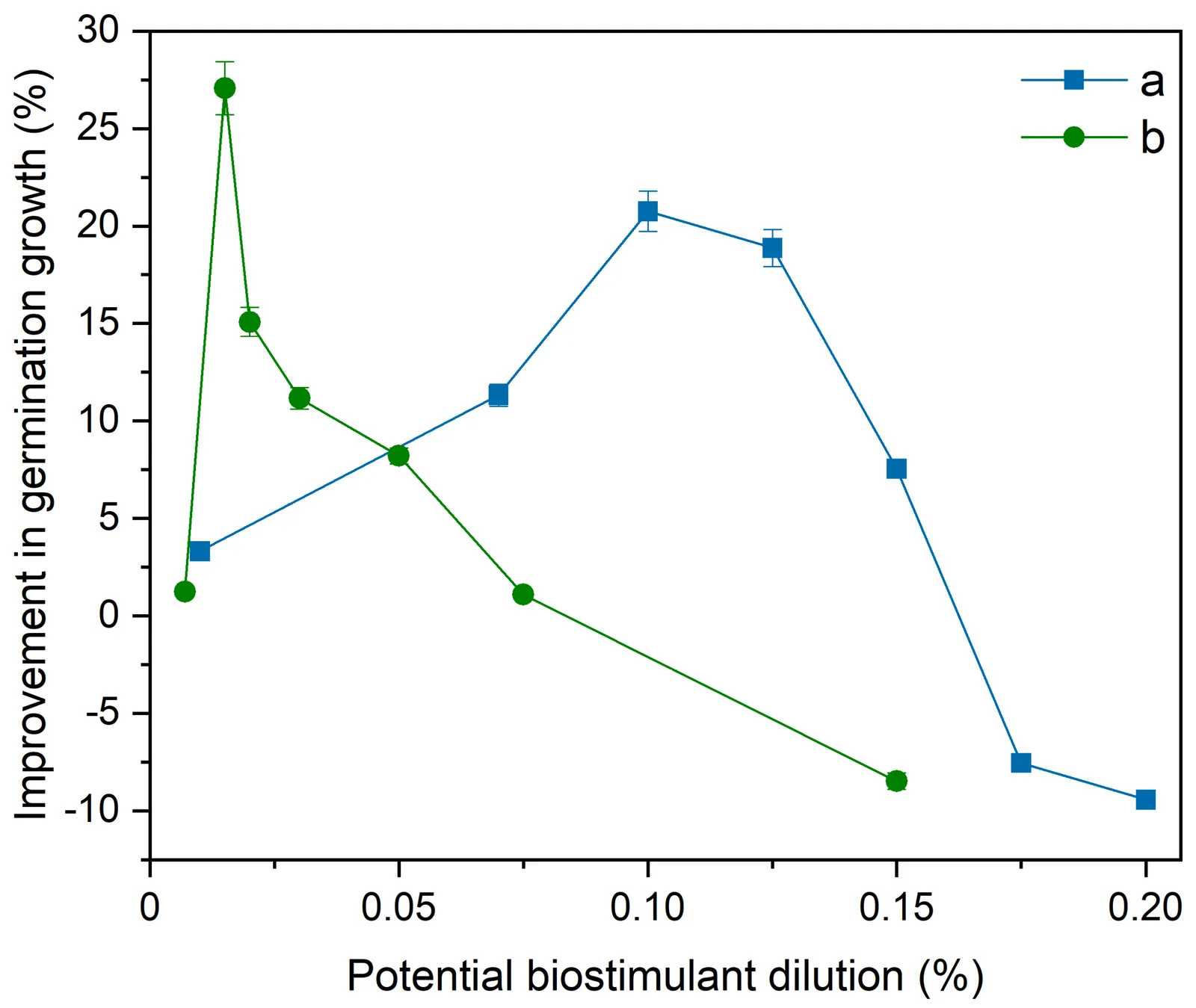
Comparison of the improvement in growth by germination of Chinese cabbage seeds with a hydrolysed product from (a) 1.5% ALC, 1% PAN from unhairing–liming wastewater and (b) 1.0% ALC, 1% PAN from lime-fleshing wastewater as a function of dosage of the potential biostimulant evaluated.

To evaluate the growth-promoting potential of the developed hydrolysates, in vitro germination assays were carried out using cabbage seeds, following a methodology commonly applied for the preliminary screening of hydrolysed protein with growth-stimulating activity [47]. The hydrolysates obtained from unhairing–liming wastewater and lime-fleshing wastewater were applied at concentrations ranging from 0.01% to 0.20%. This concentration range was selected to identify the maximum dilution at which the amino acid-based hydrolysates maximised early seed growth without causing phytotoxicity or osmotic inhibition. The results are shown in Figure 6.

The results presented in Figure 6 show that both protein hydrolysates produced a clear growth-stimulating effect on Chinese cabbage seed germination, although the magnitude of the response depended on the origin of the hydrolysate and on the applied concentration. The hydrolysate obtained from unhairing–liming wastewater increased germination growth by up to 20.7% at a dilution of 0.10%. In contrast, the hydrolysate produced from lime-fleshing wastewater achieved a higher maximum improvement, reaching 27.1% at a lower dilution of 0.07%. This indicates that the lime-fleshing wastewater hydrolysate was more effective at lower application rates, which is consistent with its higher concentration of total and free amino acids.

The stronger response observed for the lime-fleshing wastewater hydrolysate can be explained by its composition. This hydrolysate contained a higher total amino acid content than the unhairing–liming wastewater hydrolysate, 52.4% compared with 34.8%, and also a higher free amino acid content, 17.0% compared with 11.4%. Since free amino acids are directly available for plant uptake and can rapidly participate in metabolic pathways, a hydrolysate richer in free amino acids is expected to exert a stronger growth-stimulating effect at lower doses. In addition, the amino acid profile of each hydrolysate also plays a decisive role. The lime-fleshing wastewater hydrolysate contained higher proportions of glycine and arginine in the total amino acid fraction than the hydrolysate obtained from unhairing–liming wastewater. These amino acids are particularly relevant for plant metabolism. Arginine is involved in nitrogen storage and transport and can act as a precursor of polyamines and nitric oxide-related pathways, which are associated with chlorophyll synthesis, nutrient uptake and stress signalling [42]. Glycine is also involved in chlorophyll formation, photorespiration and metabolic regulation. Therefore, the higher abundance of these amino acids may partly explain the greater stimulation observed for the lime-fleshing wastewater hydrolysate.

The statistical analysis indicated that both the type of hydrolysate and the applied potential biostimulant concentration significantly affected seed germination and growth (*p* < 0.05). The two-way ANOVA with interaction, followed by the LSD post-hoc test at a 95% confidence level, revealed statistically differentiated groups, supporting the differences observed among treatments. In both hydrolysates, the response followed a dose-dependent pattern characterised by an initial increase in germination growth at low concentrations, followed by a maximum response and a subsequent decline at higher doses. This behaviour confirms that the growth-stimulating effect of protein hydrolysates is strongly concentration-dependent and that an excessive dose may reduce or even inhibit seed growth.

The decline observed at concentrations above the maximum range can be attributed to several complementary mechanisms. First, protein hydrolysates obtained from tannery wastewater streams contain not only peptides and free amino acids, but also residual salts and other compounds derived from the original effluents and from the hydrolysis process. Chinese cabbage seeds are sensitive to both bioactive compounds and residual chemicals, such as salts, acids and bases, which may remain in hydrolysate-based products after processing [48,49]. At excessive concentrations, the ionic load of the hydrolysate may increase the osmotic pressure of the germination medium, reducing water uptake by the seed and limiting radicle elongation. This salt-induced osmotic stress is a plausible explanation for the inhibition observed at the highest tested concentrations.

Second, high concentrations of free amino acids may disturb the nutritional balance of the germination medium. Although amino acids are beneficial at low or moderate doses, excessive amounts may no longer be efficiently absorbed or metabolised by the plant. Instead, they may alter the pH and ionic equilibrium of the medium, interfering with essential physiological processes during early growth. Chinese cabbage grows optimally within a pH range of approximately 5.8–7.9 [50], and deviations from this range may negatively affect germination and root development. In this context, excess amino acids and accompanying salts may contribute to local pH changes or osmotic imbalance, reducing the stimulatory effect of the hydrolysate [51].

Third, the inhibitory effect observed at high hydrolysate concentrations may also be related to the specific composition of the amino acid mixture. Previous studies have shown that excessive concentrations of some amino acids, including phenylalanine, valine and proline, can negatively affect plant growth [52]. This suggests that the hydrolysed protein with a growth-stimulating effect does not depend only on the total amount of amino acids supplied but also on the balance among individual amino acids. Therefore, the decrease in germination growth at concentrations above the optimum may result from the combined effect of osmotic stress, ionic strength, pH modification and excess of specific amino acids.

Taken together, the germination assays demonstrate that the hydrolysates obtained from both unhairing–liming wastewater and lime-fleshing wastewater exerted growth-stimulating activity under controlled germination conditions, supporting their potential application as amino acid-based biostimulant products. However, the lime-fleshing wastewater hydrolysate showed a stronger effect at a lower dose, plausibly due to its higher free amino acid content and its amino acid profile enriched in arginine and glycine. The unhairing–liming wastewater hydrolysate also produced a significant improvement in seed growth, confirming that this effluent can be valorised as a source of bioactive hydrolysates, although its maximum dose was slightly higher. These findings support the use of enzymatic hydrolysis as an environmentally friendly strategy to transform tannery wastewater streams into value-added potential biostimulant products, expanding the range of raw materials currently used for the development of agricultural biostimulants [53].

## 4. Conclusions

This study demonstrates the technical feasibility at laboratory scale of converting two protein-rich tannery wastewater streams, unhairing–liming wastewater and lime-fleshing wastewater, into value-added protein hydrolysates with potential applications as free amino acid-based plant biostimulants. The results show that these effluents should not be considered only as high-load wastewaters requiring treatment but also as secondary raw materials containing recoverable collagen- and keratin-derived protein fractions. This approach is particularly relevant for the leather sector, where large volumes of wastewater and proteinaceous residues are generated during beamhouse operations.

The sequential enzymatic strategy based on Alcalase followed by Pancreatin was effective for both effluents, although the optimal conditions depended on the wastewater composition. Unhairing–liming wastewater required 1.5% Alcalase followed by 1.0% Pancreatin, yielding 70.76% hydrolysate, 80.87% protein recovery and 11.42% free amino acids on a dry matter basis. Lime-fleshing wastewater was more readily hydrolysed and reached higher values with a lower Alcalase loading of 0.7%, achieving 98.42% yield, 92.19% protein recovery and 16.97% free amino acids. This different behaviour was mainly associated with the higher concentration and partial solubilisation of collagen-derived proteins in lime-fleshing wastewater, compared with the more diluted and compositionally different unhairing–liming stream.

The hydrolysates obtained from both wastewaters showed amino acid profiles of interest for potential biostimulant applications, but with differentiated functional characteristics. Lime-fleshing wastewater hydrolysate was richer in free amino acids and showed a profile enriched in glycine and arginine, whereas unhairing–liming wastewater hydrolysate contained lower amino acid levels but a higher contribution of keratin-related amino acids and mineral components. These compositional differences may result in complementary agronomic responses depending on the crop, dose and target physiological effect.

Germination assays indicated the growth-stimulating potential of the hydrolysates. The lime-fleshing wastewater hydrolysate produced a stronger effect at a lower dose, while the unhairing–liming wastewater hydrolysate also improved seed growth, although its optimal dose was slightly higher. These findings support the use of enzymatic hydrolysis as a mild valorisation strategy for tannery wastewater streams with different origins and compositions, expanding the range of secondary raw materials that can potentially be used for the development of agricultural biostimulants.

The present study has some limitations, as the experimental work was conducted at the laboratory scale using wastewater samples obtained from a specific tannery process, while the agronomic evaluation was limited to a germination assay with Chinese cabbage. Further studies using additional wastewater batches and more representative plant growth conditions would, therefore, be required to confirm the reproducibility and broader applicability of the results. In addition, a techno-economic assessment will be necessary to determine the feasibility of implementing the proposed process at an industrial scale. Compared with conventional treatment approaches focused mainly on pollutant removal, the proposed process offers the additional possibility of recovering the protein fraction as a value-added product. Nevertheless, a quantitative environmental assessment, including energy consumption and life-cycle impacts, would be required to determine its environmental performance in comparison with current wastewater management strategies.

## Figures and Tables

**Table 1:** Summarises the proximate composition of the unhairing–liming wastewater evaluated in this study.

Parameter	Unhairing–Liming Wastewater	Lime-Fleshing Wastewater
pH	9.63 ± 0.05	6.15 ± 0.05
Solid content (%)	4.67 ± 0.37	11.08 ± 0.78
Moisture (%)	95.33 ± 1.12	88.92 ± 1.84
Fat content (%)	0.35 ± 0.08/7.49 *	0.73 ± 0.18/6.59 *
Ash content (%)	2.21 ± 0.33/47.41 *	1.25 ± 0.14/11.28 *
Total amino acid content (%)	1.55 ± 0.24/33.2 *	9.10 ± 0.85/82.13 *

* Determination in dry.

**Table 2:** Elemental analysis of unhairing–liming wastewater.

Elemental Analysis (mg/kg):	Lime-Fleshing Wastewater	Unhairing–Liming Wastewater
P	272.6	262.51
Ca	888.2	4171.00
K	40.5	43.07
Mg	364.4	7367.54
Al	108.32	123.83
Na	483.4	2744.34
Cl	367.2	387.3
Cd	<1	<1
Fe	86.5	118.87
Ni	<1	2.76
Pb	<1	<1
Cr (VI)	<1	<1
Cr total	<1	13.66
Cu	<1	<1
Zn	<1	58.71
Hg	<0.15	<0.15
As	<1	<2

Values with a symbol of less than (<) are given as a guideline, as they are below the limit of quantification.

**Table 3:** Working ranges for enzymes selected to carry out the double enzymatic hydrolysis.

Enzyme	Activity	pH	Temperature (°C)
Alcalase	*Endo*	8–9	65–70
Bioprotease	*Endo*	8–10	50–60
NP	*Endo*	11–13	50–60
Pancreatin	*Exo*	6–8	40–50

**Table 4:** Comparative performance of the different sequential enzymatic treatments applied to unhairing–liming wastewater, expressed as hydrolysate yield, protein content, free amino acid content and protein recovery.

% Enzymes	Yield (%)	Protein Content (%) *	Free AA (%) *	Protein Recovery (%)
2% ALC/1% PAN	62.54 ± 2.12	34.96 ± 2.03	7.66 ± 0.88	67.86 ± 2.17
1.5%ALC/1% PAN	70.76 ± 1.95	34.82 ± 1.95	11.42 ± 1.03	80.87 ± 1.98
1.5% BPT/1% PAN	46.29 ± 1.98	29.73 ± 2.11	6.48 ± 0.92	48.36 ± 2.07
2% BPT/1% PAN	52.37 ± 2.16	33.94 ± 1.95	7.33 ± 0.98	54.93 ± 2.09
1.5% NP/1% PAN	63.32 ± 2.05	33.54 ± 1.93	7.92 ± 1.03	65.43 ± 2.06
2% NP/1% PAN	55.33 ± 1.98	31.95 ± 2.01	7.21 ± 0.94	58.67 ± 1.93

* In dry matter.

**Table 5:** Comparative performance of the different sequential enzymatic treatments applied to lime-fleshing wastewater, expressed as hydrolysate yield, protein content, free amino acid content and protein recovery.

% Enzymes	Yield (%)	Protein Content (%) *	Free AA (%) *	Protein Recovery (%)
1.0% ALC/1% PAN	94.06 ± 2.02	58.51 ± 2.05	16.28 ± 0.93	74.68 ± 1.83
1.5% ALC/1% PAN	79.82 ± 1.77	57.78 ± 1.98	12.22 ± 0.98	67.32 ± 2.02
1.0% BPT/1% PAN	86.66 ± 1.92	53.50± 2.14	15.13 ± 1.01	56.45 ± 1.79
1.5% BPT/1% PAN	88.52 ± 1.98	52.30 ± 2.09	14.62 ± 0.91	58.28 ± 1.72
1.0% NP/1% PAN	84.23 ± 2.05	51.39 ± 1.96	12.66 ± 1.01	52.23 ± 2.12
1.5% NP/1% PAN	80.66 ± 2.01	50.67 ± 1.84	11.88 ± 0.95	50.19 ± 2.08

* In dry matter.

**Table 6:** Yield, amino acid composition and protein recovery of hydrolysates obtained from unhairing–liming wastewater using different ALC enzyme loadings in the sequential ALC/PAN process, including standard deviations (SDs).

ALC (%)	PAN (%)	Yield (%)	Total Amino Acids (%) *	Free Amino Acids (%) *	Protein Recovery (%) *
0.5	1.0	58.12 ± 1.97	39.77 ± 2.11	5.90 ± 0.85	72.40 ± 2.05
1.0	1.0	81.50 ± 2.12	37.22 ± 1.97	7.12 ± 0.91	91.10 ± 2.02
1.25	1.0	76.48 ± 2.06	31.23 ± 2.04	7.51 ± 0.93	82.63 ± 1.88
1.5	1.0	70.76 ± 1.95	34.82 ± 1.95	11.42 ± 1.03	80.87 ± 1.98
1.75	1.0	68.55 ± 2.07	32.05 ± 2.01	9.48 ± 0.93	77.98 ± 2.11
2.0	1.0	62.54 ± 2.12	34.96 ± 2.03	7.66 ± 0.88	67.86 ± 2.17

* In dry matter.

**Table 7:** Yield, amino acid composition and protein recovery of hydrolysates obtained from lime-fleshing wastewater using different ALC enzyme loadings in the sequential ALC/PAN process, including standard deviations (SDs).

ALC (%)	PAN (%)	Yield (%)	Total Amino Acids (%) *	Free Amino Acids (%) *	Protein Recovery (%) *
0.5	1.0	72.03 ± 1.81	50.87 ± 2.13	12.46 ± 0.83	52.66 ± 1.66
0.7	1.0	98.42 ± 1.97	52.39 ± 2.11	16.97 ± 0.98	92.19 ± 1.92
1.0	1.0	94.06 ± 2.02	58.51 ± 2.05	16.28 ± 0.93	74.68 ± 1.83
1.25	1.0	83.69 ± 1.84	59.69 ± 2.18	14.10 ± 0.94	71.81 ± 1.84
1.5	1.0	79.82 ± 1.77	57.78 ± 1.98	12.22 ± 0.98	67.32 ± 2.02

* In dry matter.

**Table 8:** Comparison of the RD 1009/2019 parameters corresponding to the different hydrolysed products obtained from the different tannery wastewaters evaluated.

Parameter	Hydrolysed Protein with Growth-Stimulating Activity Obtained from Unhairing–Liming Wastewater	Hydrolysed Protein with Growth-Stimulating Activity Obtained from Lime-Fleshing Wastewater
Total nitrogen (% dry matter)	6.12 ± 0.24	7.29 ± 0.24
Ammonia nitrogen (% dry matter)	0.52 ± 0.04	0.56 ± 0.06
Organic nitrogen (%dry matter)	5.22 ± 0.29	5.13 ± 0.38
Urea nitrogen (%dry matter)	0.009 ± 0.002	0.008 ± 0.003
Total amino acids (%dry matter)	34.82 ± 1.95	52.39 ± 2.11
Free amino acids (% dry matter)	11.42 ± 1.03	16.97 ± 0.98
Dry matter (%)	48.52 ± 2.88	67.86 ± 2.67
Moisture (%)	51.48 ± 2.88	32.14 ± 2.67
Organic matter (%)	43.72 ± 2.42	64.36 ± 2.34
Ashes (%dry matter)	38.27 ± 2.05	24.56 ± 1.96
Density (kg/L)	1.16 ± 0.01	1.22 ± 0.01
pH	5.82 ± 0.05	5.77 ± 0.05

## Data Availability

The data presented in this study are available on request from the corresponding author.
